# In Silico Discovery and Sensory Validation of Umami Peptides in Fermented Sausages: A Study Integrating Deep Learning and Molecular Modeling

**DOI:** 10.3390/foods14142422

**Published:** 2025-07-09

**Authors:** Haochen Geng, Chunming Xu, Huijun Ma, Youxu Dai, Ziyou Jiang, Mingyue Yang, Danyang Zhu

**Affiliations:** 1 School of Computer and Artificial Intelligence, Beijing Technology and Business University, Beijing 100048, China; fredgeng54@gmail.com (H.G.); mahuijun@th.btbu.edu.cn (H.M.); yoyo109210@163.com (Z.J.); luckydogbill@163.com (M.Y.); 2 School of Light Industry Science and Engineering, Beijing Technology and Business University, Beijing 100048, China; 18280353127@163.com; 3 School of Languages and Communication, Beijing Technology and Business University, Beijing 100048, China; 2214020439@st.btbu.edu.cn

**Keywords:** deep learning, molecular docking, umami peptides, peptide prediction

## Abstract

Deep learning has great potential in the field of functional peptide prediction. This study combines metagenomics and deep learning to efficiently discover potential umami peptides in fermented sausages. A candidate peptide library was generated using metagenomic data from fermented sausages, an integrated deep learning model was constructed for prediction, and SHAP (SHapley Additive exPlanations) interpretability analysis was performed to elucidate the key amino acid features and contributions of the model in predicting umami peptides, screening the top ten peptides with the highest predicted probability. Subsequently, molecular docking was performed to assess the binding stability of these peptides with the umami receptor T1R1/T1R3, selecting the three peptides DDSMAATGL, DGEEDASM, and DEEEVDI with the most stable binding for further study. Docking analysis revealed the important roles of the key receptor residues Glu301, Arg277, Lys328, and His71 in hydrogen bond formation. Molecular dynamics simulations validated the robust integrity of the peptide–receptor associations. Finally, sensory evaluation demonstrated that these three peptides possessed significant umami characteristics, with low umami thresholds (0.11, 0.37, and 0.44 mg/mL, respectively). This study, based on metagenomics and deep learning, provides a high-throughput strategy for the discovery and validation of functional peptides.

## 1. Introduction

Umami is the fifth basic taste sensation following sweet, sour, bitter, and salty [1]. The taste-presenting properties of umami derive from the synergistic effects of free amino acids, flavor nucleotides, organic acids, and umami peptides [2], with umami peptides being the core taste-presenting factors. These active peptides are widely found in systems of microbial interaction with food matrices, in fermented foods such as soy sauce and miso [3], or enzymatic products of cereal proteins such as soybean and wheat [4]. Fermented sausage, as a meat fermentation product, undergoes a synergistic effect of microbial metabolism and protein degradation during processing, releasing free amino acids and nucleotides, thus producing umami peptides with unique structures. Although direct research on umami peptides in fermented sausages is currently relatively limited, significant progress has been made in the study of fermented hams. For instance, Dang et al. [5] identified two umami-active peptides, CCNKSV and AHSVRFY, from Jinhua ham and Parma ham. Furthermore, Cui et al. [6] isolated and identified four umami peptides from Xuanwei ham, with sequences of MDAIKKMQ, RKYEEVAR, YVGDEAQSKRG, and VNVDEVGGEALGR. Considering that both fermented hams and fermented sausages are fermented meat products, and their flavor formation processes involve similar microbial metabolism and protein degradation, the findings and identification results of umami peptides in fermented hams can serve as an important reference for predicting and exploring potential umami peptides in fermented sausages. Consequently, fermented sausage can be regarded as a source of umami peptides.

The conventional approach for identifying umami peptides involves protein hydrolysis, followed by separation and purification using chromatography and mass spectrometry techniques. Ultimately, the confirmation of their freshness activity is achieved through artificial sensory evaluation. Liu et al. [7] screened seven umami peptides by protein hydrolysis. In a separate study, Chen et al. [8] identified five umami peptides by combining continuous enzymatic hydrolysis with ultrafiltration technology. However, this approach involves complicated separation and purification steps and relies on manual subjective determination, which is inefficient and expensive, and it is difficult to meet the demands of high-throughput screening.

In recent years, the rise of artificial intelligence techniques such as machine learning and deep learning has significantly boosted the field of umami peptide prediction. Jiang et al. [9] proposed the iUmami-DRLF model, which combines mLSTM-based UniRep feature extraction with logistic regression. Charoenkwan et al. [10] predicted umami peptides by developing the iUmami-SCM model using a scorecard approach combined with dipeptide propensity scores. Jiang et al. [11] developed the iUP-BERT model, which used the bidirectional encoder converter BERT to extract peptide sequence features. Cui et al. [12] predicted umami peptides using the gradient boosting decision tree model Umami_YYDS, which was validated by sensory experiments. Yue et al. [13] proposed the Umami-MRNN model combining a recurrent neural network (RNN) and multilayer perceptron (MLP). Hansan et al. [14] developed the UMPred-FRL model by integrating seven feature encodings using a feature learning approach. The UmamiPreDL model [15] integrated ProtBERT’s protein sequence comprehension with CNN-driven local feature extraction, thereby demonstrating the feasibility of machine learning and deep learning in umami peptide discovery.

A key advantage of artificial intelligence lies in its ability to automatically extract complex hidden patterns from peptide sequences, thereby overcoming the reliance of traditional methods on manually designed molecular descriptors. The model integrates multi-source feature encoding and dynamic feature extraction, enabling the effective analysis of long-range dependence and spatial distribution between amino acid residues. Additionally, it addresses the issue of small-sample imbalance through data enhancement technology, enhancing the model’s generalization capability despite limited datasets. Compared with traditional hydrolysis purification methods, artificial intelligence methods help to predict umami peptides more accurately and effectively, making the high-throughput screening of novel umami peptides possible.

Protein structure prediction methods, such as AlphaFold, can aid in determining the three-dimensional shape of a target protein from its amino acid sequence. Molecular docking analyses involve simulating interactions between a ligand and a receptor to predict binding properties. The human perception of umami flavors occurs via the T1R1/T1R3 taste receptors, located on the anterior aspect of the tongue [16]. In recent umami peptide studies, two complementary computational approaches were systematically integrated by Dang et al. [17] for prediction. Amin et al. [18] simulated binding patterns through LC-MS/MS technology combined with molecular docking. Wang et al. [19], through amino acid distribution and active site analysis, proposed a new decision rule based on a scorecard to predict umami peptides. Chang et al. [20] predicted the binding mode of the umami peptide EGTAG using this method. Ruan et al. [21] predicted five umami peptides by analyzing their binding properties to the receptor using hydrogen bonding and hydrophobic interactions. These studies not only validate the reliability of the methods of homology modeling and molecular docking in the prediction of umami peptides but also lay the methodological foundation for the design of flavor molecules.

However, extant models predominantly depend on a solitary infrastructure for prediction, which is susceptible to suboptimal accuracy due to one-sided feature capture, faces challenges in balancing local sequence features and long-range dependencies, and may overlook the cross-scale association of peptide–receptor dynamic binding. In this study, we propose an integrated deep learning framework that integrates CNN, Transformer, LSTM, and Attention architectures for umami peptide prediction and ultimately integrates multidimensional information to improve the resulting model’s robustness and generalization ability. In addition, to gain a deeper understanding of the model’s prediction mechanism, we employed the SHapley Additive exPlanations (SHAP) analysis method. Through SHAP analysis, we were able to perform global interpretation to identify the amino acid positions that have the greatest impact on umami prediction; provide local explanations that clarify why specific peptide segments are predicted as umami peptides; and reveal the positive or negative contributions (i.e., feature directions) of specific amino acids at specific positions.

## 2. Materials and Methods

### 2.1. Sample Source

Based on the research method of Ilario et al. [22], the sausage was made using pork (77%), pork fat (23%), and salt (2.9%), as well as spices, sucrose, and wine as raw materials. Some sausages were supplemented with commercial starter cultures; then, the meat mixture was stuffed into casings and fermented and matured under climate-controlled conditions. The specific temperature and relative humidity profiles for the heating, fermentation, and ripening phases are detailed in Table 1. The control group sausages were naturally fermented under the same environmental conditions as the experimental group but without the addition of commercial starter cultures. For subsequent analysis, equal portions were taken from both the inoculated group and the naturally fermented group prior to filling (Day 0) and on Day 3, Day 7 (during fermentation), and Day 40 (after ripening). For each sampling point, three sausages were sampled, and from each sausage, samples were taken from the top, middle, and bottom to ensure randomness. These sampling points were chosen to cover the initial state for comparison (Day 0), the critical early and mid-fermentation stages where flavor compounds begin to form (Day 3 and Day 7), and the stage of the final ripened product where flavor has stabilized (Day 40).

### 2.2. Genetic Sequencing and Data Analysis

The sequencing method was based on that of Ilario et al. [22] and improved. Specifically, after extracting and purifying total DNA, the Nextera XT library preparation kit (Illumina, San Diego, CA, USA) was used to fragment the DNA sequence library and label sequencing adapters according to the manufacturer’s instructions. The library was then quantified, and the quality and size distribution of the library were assessed. Sequencing was performed on the Illumina MiSeq system (Illumina, San Diego, CA, USA) using a 151-cycle paired-end run mode, processing 4 samples per run. The final output was raw data formatted as FASTQ files.

### 2.3. Construction of the Peptide Dataset

**Figure 1:**
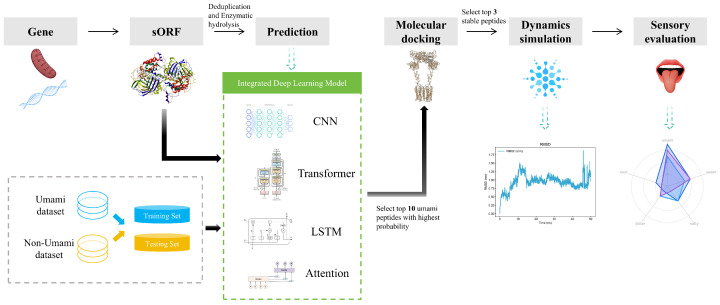
Study workflow.

The study workflow is shown in Figure 1. This study selected approximately 150,000 sequencing data and predicted protein open reading frames using the getORF module of the EMBOSS toolkit (version 6.5.7) [23]. The Protein Digestion Simulator tool (version 2.4.8937) (https://pnnl-comp-mass-spec.github.io/Protein-Digestion-Simulator/) (accessed on 7 July 2025) simulated the combined enzymatic hydrolysis of trypsin and chymotrypsin, generating peptide products close to real experimental conditions. The CD-HIT tool (version 4.8.1) [24,25] was used to perform redundancy removal on the digested peptide segments, setting a 90% sequence similarity threshold, ultimately obtaining a non-redundant peptide segment set as the foundational data for deep learning model training and functional analysis.

### 2.4. Prediction Model Design and Validation

In order to improve the model performance, based on previous research [26] on the impact of label data balance and reliability on model performance, a balanced training set containing 254 umami peptides and 254 non-umami peptides was constructed at a 1:1 ratio. The umami peptide data were sourced from previous research [4,7,10,12,13,14,18,19,20,21,27] and verified umami peptides from the BIOPEP-UWM database [28]; the non-umami peptides were selected from negative samples reported in [10,11,12,13,14]. Through strict sequence redundancy removal, the uniqueness and integrity of the dataset were effectively ensured, thereby guaranteeing the reliability of the training data and its support for the model’s predictive performance.

In this study, four deep learning models—CNN, Transformer, LSTM, and Attention—were used to classify and predict umami peptides. CNN captures short-range features of local amino acid sequences through convolutional kernels to identify motifs related to umami [29]; Transformer integrates global contextual information through self-attention mechanisms to explore deep sequence correlations [30]; LSTM models long-range dependencies of peptide chains based on gating mechanisms to analyze dynamic sequence patterns [31]; and Attention focuses on the weight distribution of key amino acid residues to enhance feature interpretability [32]. These four models construct complementary features from dimensions such as local patterns, temporal dynamics, key targets, and global semantics, ultimately forming an integrated prediction framework that significantly improves the accuracy and generalization ability of analyzing the spatial structure–activity relationship of umami peptides.

The peptide sequence is first passed through the input layer and processed by the four sub-models, each extracting local features and long-range dependencies within the peptide sequence. Each model independently performs classification prediction, and the final prediction results of the four models are integrated through the output layer. Only when all models unanimously agree that the peptide is an umami peptide is it finally determined to be an umami peptide. This ensemble strategy effectively improves the classification accuracy and reduces the occurrence of false positive predictions. To construct the final umami peptide screening model, we randomly select 80% of positive and negative samples for model development. The remaining 20% are reserved for model assessment. The model’s performance is gauged using the following equations, where Pr represents precision, Rc represents recall, Sp represents specificity, ACC represents accuracy, and *F*1 represents the *F*1 score.

(1) $$Pr=\frac{TP}{TP+FP}$$

(2) $$Rc=\frac{TP}{TP+FN}$$

(3) $$Sp=\frac{TN}{TN+FP}$$

(4) $$ACC=\frac{TP+TN}{TP+FN+TN+FP}$$

(5) $$F1=\frac{2\times Pr\times Rc}{Pr+Rc}$$

In performance evaluation, TN (True Negative), FN (False Negative), TP (True Positive), and FP (False Positive) are standard metrics. Once the model’s performance is confirmed, it then predicts umami peptides in new unknown sequences, providing a probability value for each prediction.

### 2.5. Model Interpretability Analysis

To further deepen the understanding of the model’s prediction mechanism for umami peptides and enhance the interpretability of the prediction results, we employ the SHAP (SHapley Additive exPlanations) analysis method. SHAP analysis is performed with the aim of quantifying the contributions of amino acid types at specific positions in the peptide chain to the model’s prediction results, providing local explanations for why a particular peptide segment is predicted as an umami peptide, quantifying whether a specific amino acid at a specific position contributes positively or negatively, thereby revealing feature direction; simultaneously, through global explanations, it identifies the amino acid sites that have the greatest impact on umami prediction. Through interpretability analysis, we can reveal the internal workings of the model “black box” [33], providing more intuitive and quantitative evidence for understanding the sequence–activity relationship of umami peptides.

### 2.6. Discovery of Umami Peptides

Based on this model, we predicted unknown sequences and selected the top 10 target sequences ranked by umami probability as potential umami peptides. Toxicity risk assessment was conducted using the ToxinPred platform (version 1.0) [34,35]; key physicochemical parameters such as molecular weight, theoretical isoelectric point, and average hydrophilicity coefficient were analyzed using the ProtParam tool (version 1.0) on the Expasy platform [36]; and the net charge at neutral pH (7.0) was analyzed using the Protein Calculator (version 3.4) (https://protcalc.sourceforge.net/) (accessed on 7 July 2025) to provide data support for subsequent research.

### 2.7. Construction of Receptor Proteins

To construct the three-dimensional structural model of the umami receptor T1R1/T1R3, we employed protein structure prediction. First, we obtained the sequences for umami receptors T1R1 (UniProt ID: Q7RTX1) and T1R3 (UniProt ID: Q7RTX0) from the UniProt database (https://www.uniprot.org/) (accessed on 7 July 2025). Based on the acquired amino acid sequences, AlphaFold3 [37] was used to predict and build three-dimensional structural models of the receptors. Subsequently, the constructed models were visualized using molecular modeling and visualization software. To verify the stereochemical quality and structural stability of the models, we further evaluated them using Ramachandran plots, a method consistent with commonly used protein structure modeling and evaluation procedures [38].

### 2.8. Molecular Docking

The three-dimensional structures of the screening peptides were constructed computationally. Molecular docking of the umami peptides (treated as small-molecule ligands) with the T1R1/T1R3 macromolecular receptor was subsequently performed using molecular modeling software. During the preprocessing stage, structural optimization of both the receptor and ligands was conducted, including the removal of crystallographic water molecules, elimination of co-crystallized ligands, and addition of hydrogen atoms, after which the structures were saved in PDBQT format. For docking parameter settings, the ligand-binding domain was defined as a spherical region with a radius of 12.2078 centered at the coordinates X = 194.677, Y = 220.766, and Z = 123.678. Finally, we visualized and analyzed the three-dimensional spatial arrangements using PyMOL (version 3.1.4.1) (https://pymol.org/) (accessed on 7 July 2025).

### 2.9. Peptide Synthesis and Purification

This study used the solid-phase synthesis strategy developed by Krisztina et al. [39] and Carpino [40] to prepare umami peptides. Using Rink amide MBHA resin as the carrier, the resin was first swollen in DMF at 25 °C for 3 h; then, Fmoc-protected amino acids were pre-activated at 0 °C for 10 min, followed by a 3-hour coupling reaction at 25 °C with shaking at 120 rpm. After each coupling step, efficiency was monitored by the Kaiser test, alternating acetylation capping, solvent washing, and Fmoc deprotection. Following peptide chain elongation, the resin underwent lyophilization for 12 h. It was subsequently separated from the peptide over 4 h at 25 °C, employing a cleavage agent containing trifluoroacetic acid. After filtration, chilled diethyl ether was introduced to isolate the peptide. This mixture then underwent centrifugation at 10,000× *g* and 25 °C for 15 min, followed by freeze-drying to yield the raw product. Finally, reverse-phase high-performance liquid chromatography (RP-HPLC) served to purify the target peptide. A gradient elution consisting of 0.1% trifluoroacetic acid in water and acetonitrile was employed. The desired peak was collected and then lyophilized, yielding the refined peptide for further studies.

### 2.10. Molecular Dynamics Simulation

Using GROMACS 2020 [41], we conducted molecular dynamics simulations of the umami peptide–receptor complex to systematically investigate the dynamic association of the peptide with its receptor [42]. The complex was solvated in a TIP3P water model, utilizing a widely recognized molecular mechanics force field. Sodium and chloride ions were then introduced to achieve system neutrality and replicate physiological ionic concentrations. The simulation protocol unfolded in three distinct stages. First, the system underwent 2f iterations of energy reduction, employing a gradient-based approach to eliminate any atomic overlaps. This was followed by a 100-picosecond stabilization phase at 36.85 °C, maintaining a constant number of particles, volume, and temperature. Subsequently, a second 100-picosecond stabilization period was conducted at one atmospheric pressure, ensuring a constant number of particles, pressure, and temperature. These preparatory steps then led into a 50-nanosecond molecular dynamics simulation under constant temperature and pressure conditions. Trajectory analyses were performed to calculate key parameters such as the root mean square deviation (RMSD), root mean square fluctuation (RMSF), and hydrogen bond characteristics (HB).

### 2.11. Sensory Evaluation

#### 2.11.1. Panel Training

Sensory evaluation strictly followed the Amin team’s protocol [18]. The study was conducted in accordance with the Declaration of Helsinki, and the protocol was approved by the Ethics Committee of Beijing Technology and Business University (project identification code: 2025-95) on 12 March 2025. Informed consent was secured from all volunteers. A professional evaluation panel of 12 members, consisting of 6 females and 6 males, completed the tests over three days in a standard sensory laboratory with a constant temperature of 25 °C and standard lighting, ensuring data reliability through repeated tests on multiple days. The evaluators received systematic training, which encompassed triangle tests and the establishment of taste benchmarks. This was accomplished using five reference solutions: sucrose (1%) served as the standard for sweetness; sodium chloride (0.35%) for saltiness; caffeine (0.08%) for bitterness; monosodium glutamate (MSG) (0.35%) for umami; and citric acid (0.08%) for sourness. Taste intensity was quantified on a 0–10 scale, where 0 indicated a weak perception, 5 was the standard reference intensity, and 10 represented a strong perception.

#### 2.11.2. Assessment of Umami Thresholds for Synthetic Peptides

This study used a taste dilution analysis method that improved upon the experimental method published by Li et al. [43] to determine the umami threshold of synthetic umami peptides. The method proceeded as follows. First, deionized water was used as the solvent, and the synthetic peptides were diluted in a 1:1 gradient to form a concentration series. Then, sensory testing was conducted using the triangle test at a constant temperature of 25 ± 2 °C and 65% humidity. During the experiment, trained sensory panel members performed blind tests from low to high concentrations step by step. When a certain concentration group could be clearly distinguished from the blank group (deionized water) but the previous concentration group could not, the average concentration of the two groups was taken as the threshold.

#### 2.11.3. Umami Enhancement of Synthetic Peptides

Following Chang et al. [44], we studied the umami enhancement effect. A 1 mg/mL sample of the peptide was created using a 0.35% MSG solution. The enhancement intensity was evaluated on a 10-point scale (0 points for ineffective, 10 points for significant), and the enhancement threshold was determined through taste dilution analysis.

#### 2.11.4. Electronic Tongue Measurement

A prevalent method for flavor assessment involves the use of an e-tongue [45]. The e-tongue’s testing procedure was refined based on the experimental guidelines from Phat et al. [46]. The umami peptides were formulated as a 1 mg/mL solution in deionized water. Subsequent to sensor membrane potential adjustment, the testing and subsequent data evaluation were conducted utilizing the Japanese Insent TS5000Z e-tongue system (Insent, Atsugi, Japan), using MSG as a standard for data evaluation and comparison.

### 2.12. Statistical Analysis

All experimental data stemmed from three independent replicates, with the arithmetic mean serving as the final reported result. Statistical analyses were conducted using Origin 2022 software (Origin Lab, Inc., Northampton, MA, USA). Independent samples *t*-tests were used to determine statistical significance between the umami peptides and MSG. Asterisks (*) indicate significant differences: * for *p* < 0.05, ** for *p* < 0.01, and *** for *p* < 0.001.

## 3. Results and Analysis

### 3.1. Model Construction and Evaluation Results

We performed metagenomic sequencing to acquire FASTA data for microbial communities, subsequently obtaining clean sequences by applying quality control and splice trimming. In addition, we predicted open reading frames (ORFs) with the getORF program from EMBOSS, followed by enzymatic simulations by trypsin and tryptic rennet. Peptides were de-redundantly processed using CD-HIT, and finally 150,000 non-redundant peptide data were selected for subsequent prediction analysis.

The sequences were predicted using the integrated deep learning model proposed in this paper. The precision, recall, accuracy, specificity, and F1 values for the four models and overall are shown in Table 2.

In order to fully validate the performance and generalization ability of our model, we also performed a comparative analysis with other models (UMpred-FRL [14], UmamiYYDS [12], UmamiBert [47], and mlp4umami [48]). The performance of our model uses the average of the four sub-models.

**Figure 2:**
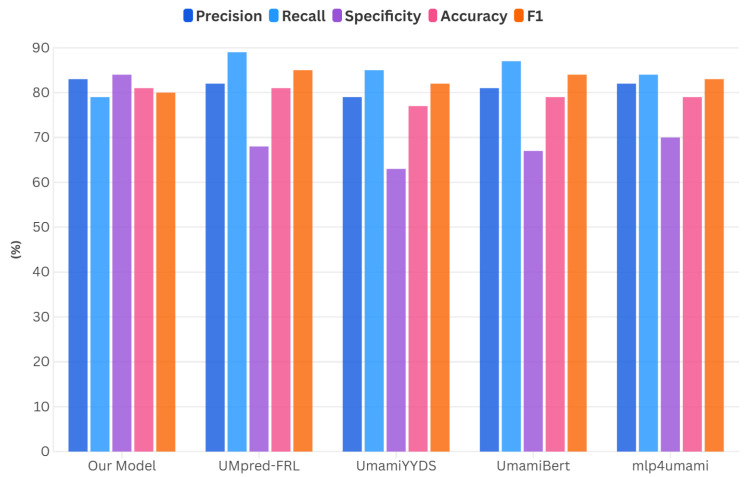
Performance comparison of different models.

Table 3 and Figure 2 show that our model demonstrates significant advantages in multiple key performance indicators. Although its recall rate was slightly lower than other models, our model performed excellently in terms of precision and specificity, significantly outperforming other comparison models (especially in specificity, which was on average 17% higher than that of the other models). This indicates that our model has a stronger ability to reduce false positives and improve prediction accuracy. At the same time, in terms of overall accuracy, our model was on par with UMpred-FRL and surpassed the other models. The above data collectively indicate that the performance metrics of this model meet the requirements for umami peptide screening and have practical application value.

### 3.2. Model Interpretability Analysis

The SHAP analysis results provide local explanations, feature directions, and global explanation information of the model through multidimensional visualization. The force plot provides a local explanation for the prediction of a single peptide segment. Starting from the model’s average prediction value, it shows how each feature collectively pushes the prediction toward the final predicted value for that specific peptide segment through stacked red (positive contribution) and blue (negative contribution) arrows. This allows us to understand why a particular peptide segment is predicted as an umami peptide; the waterfall plot clearly indicates the direction of features. Red bars represent positive contributions of the feature to the prediction, while blue bars represent negative contributions, clearly revealing the promoting or inhibiting effects of specific amino acids at specific positions and their intensities. Furthermore, the amino acid position importance plot visually displays the global impact of amino acid positions on umami prediction. The length of its bars directly reflects the average contribution of each amino acid position to the model’s prediction.

**Figure 3:**
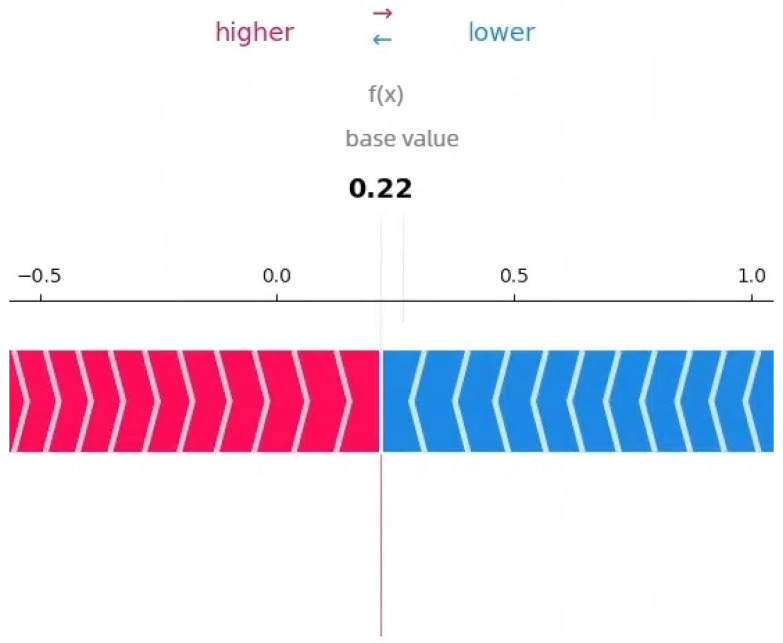
Interpretability analysis of CNN: local explanation.

**Figure 4:**
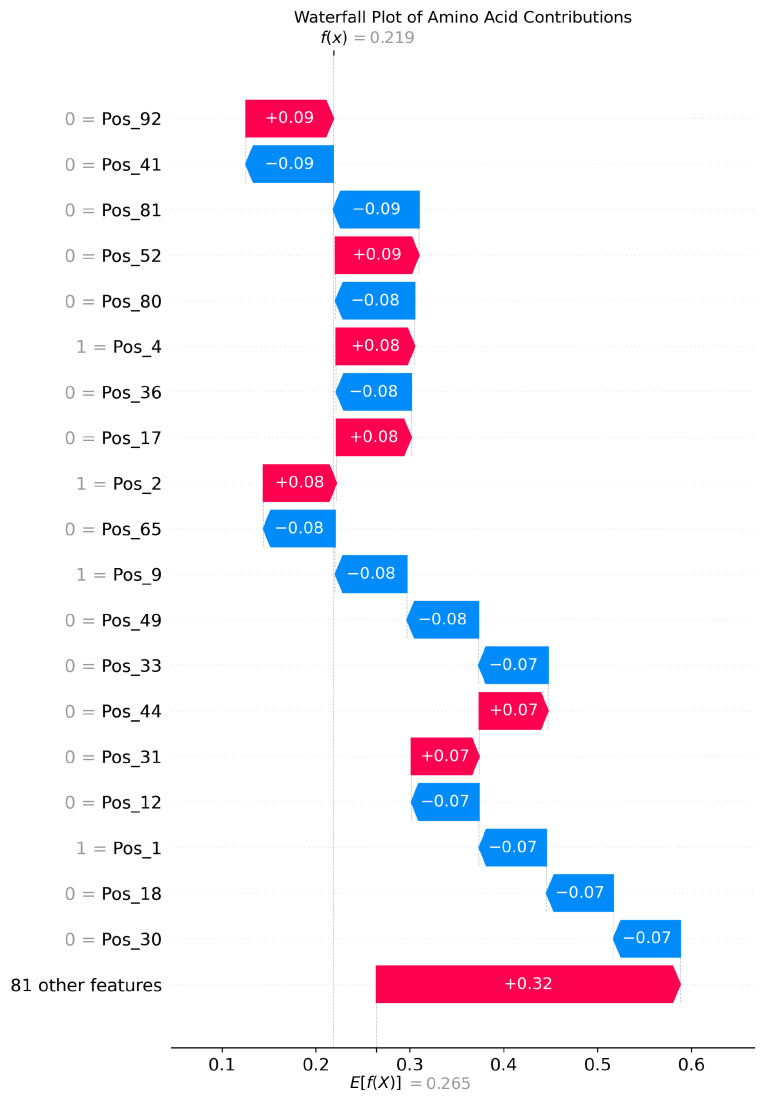
Interpretability analysis of CNN: feature direction.

**Figure 5:**
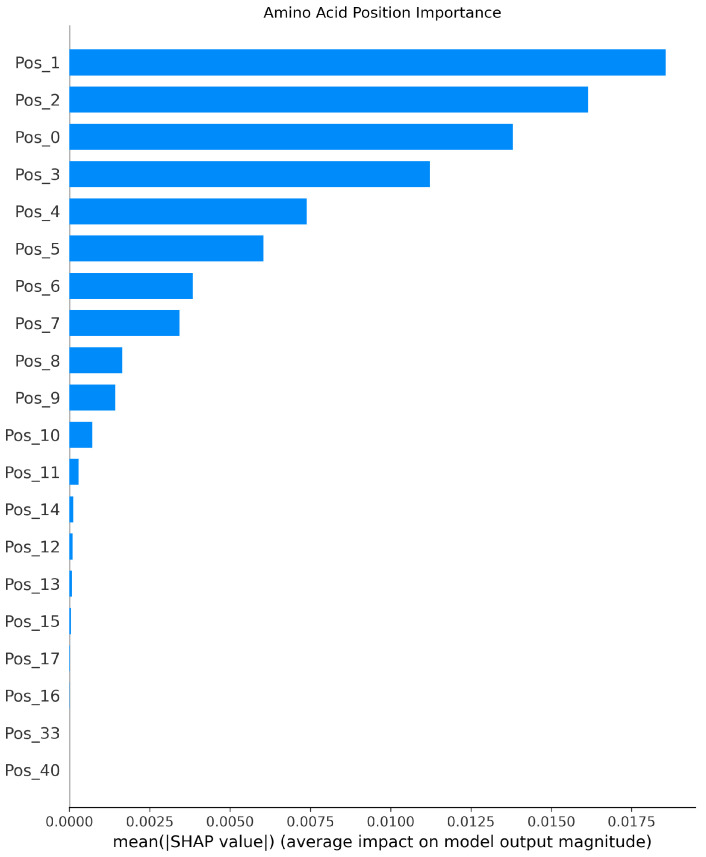
Interpretability analysis of CNN: global explanation.

Figure 3, Figure 4 and Figure 5 present the interpretability analysis of the CNN model. For local explanations, the model’s predicted value was 0.22. In terms of feature direction, the waterfall chart shows that the cumulative contribution of most features reached +0.32, indicating that the synergistic positive effects of many features have a significant impact on the prediction results. At the global explanation level, amino acids at positions Pos_1, Pos_2, and Pos_0 exhibited the highest average SHAP values, indicating that they are the most important features influencing umami prediction.

**Figure 6:**
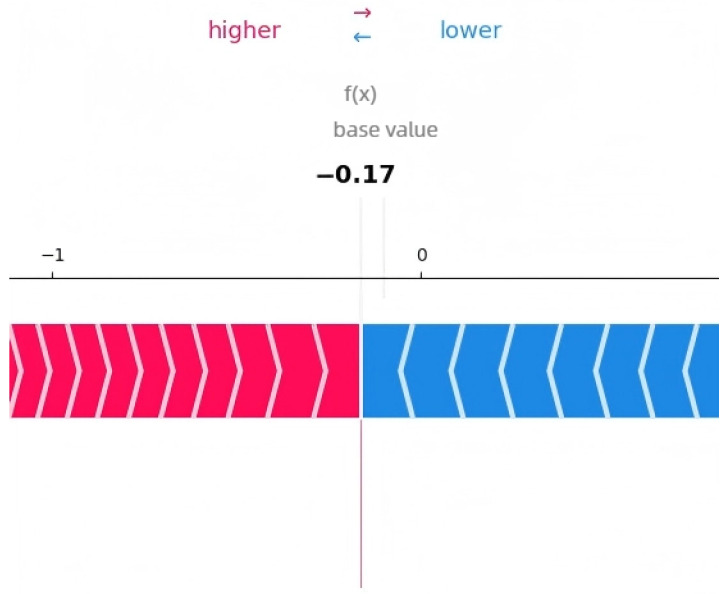
Interpretability analysis of Transformer: local explanation.

**Figure 7:**
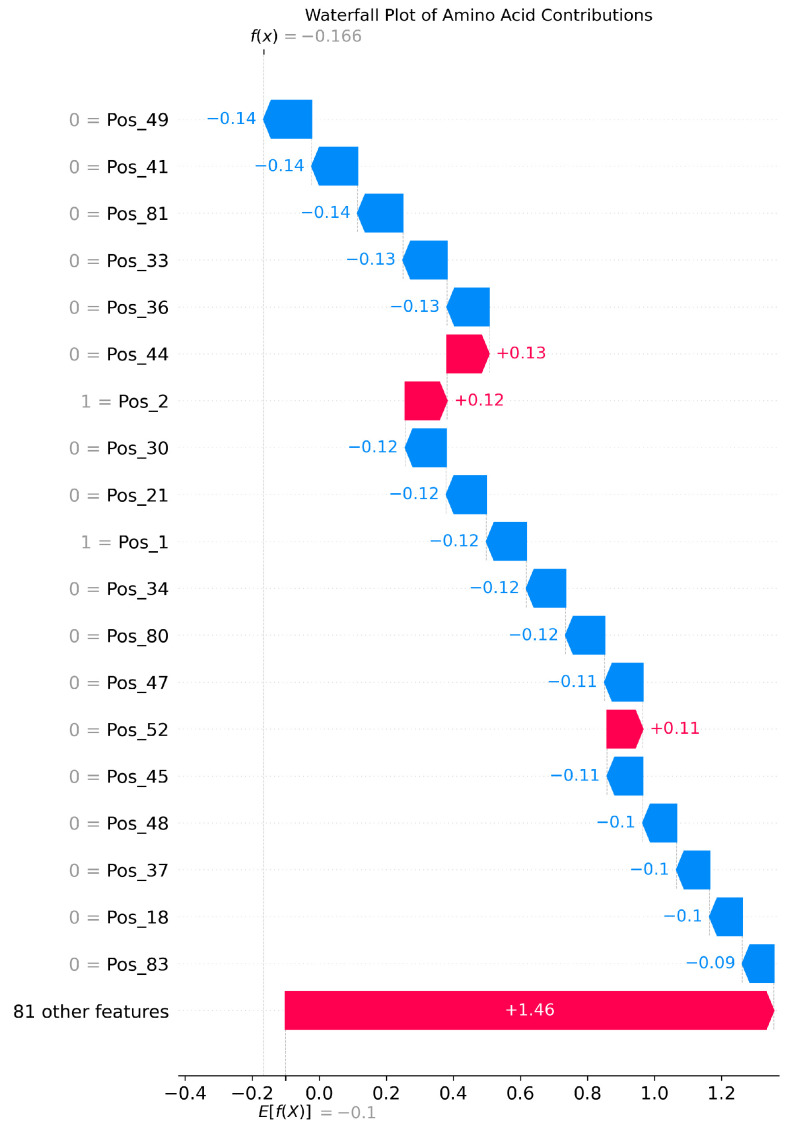
Interpretability analysis of Transformer: feature direction.

**Figure 8:**
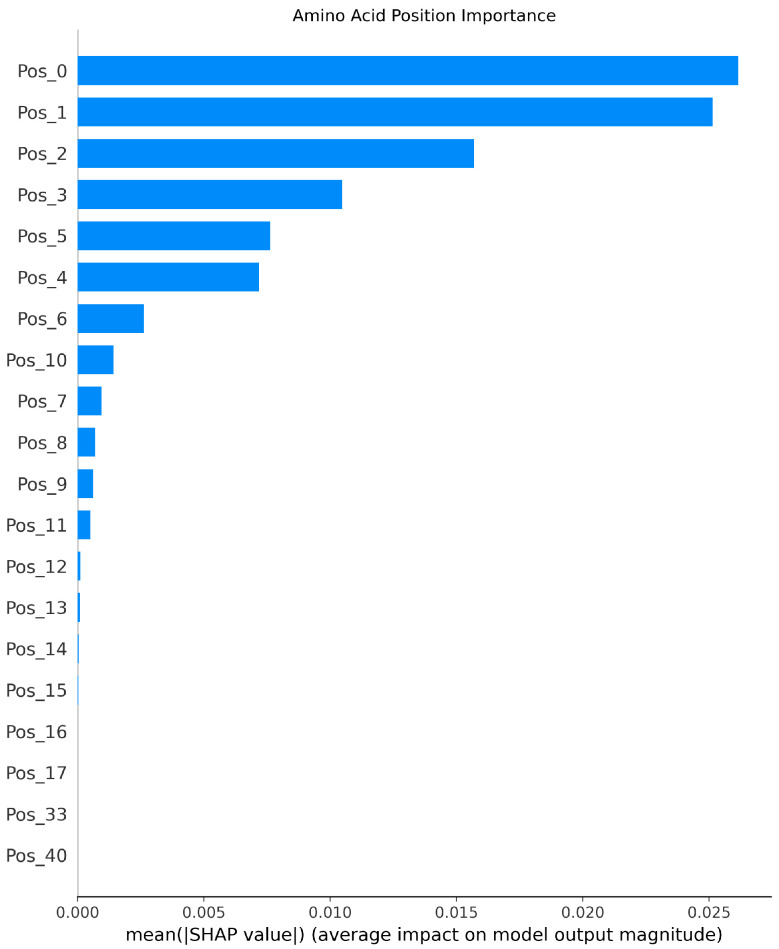
Interpretability analysis of Transformer: global explanation.

Figure 6, Figure 7 and Figure 8 present the interpretability analysis of the Transformer model. For local explanations, the model’s predicted value was −0.17. Although this is a negative contribution, the cumulative contribution of most features in the feature direction reached +1.46, indicating that the synergistic positive effect of these features has a significant impact on the prediction results. At the global explanation level, amino acids in the starting region exhibited the highest average SHAP values, indicating that they are the most important features influencing umami prediction.

**Figure 9:**
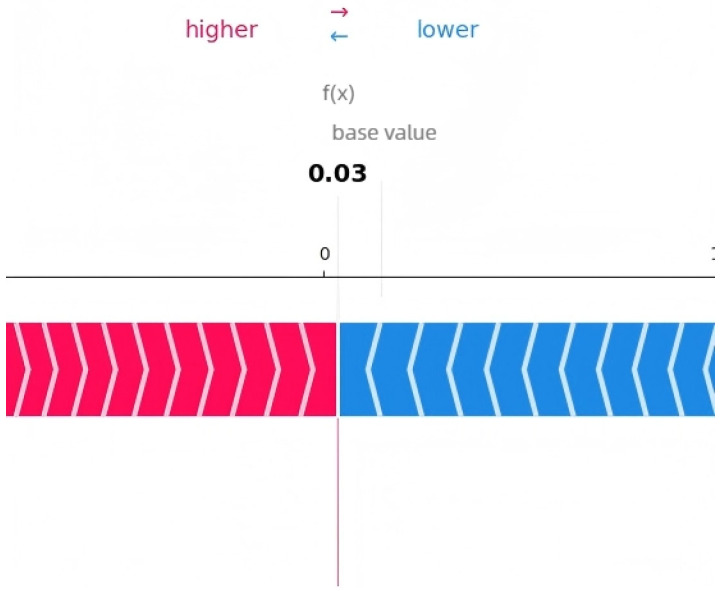
Interpretability analysis of LSTM: local explanation.

**Figure 10:**
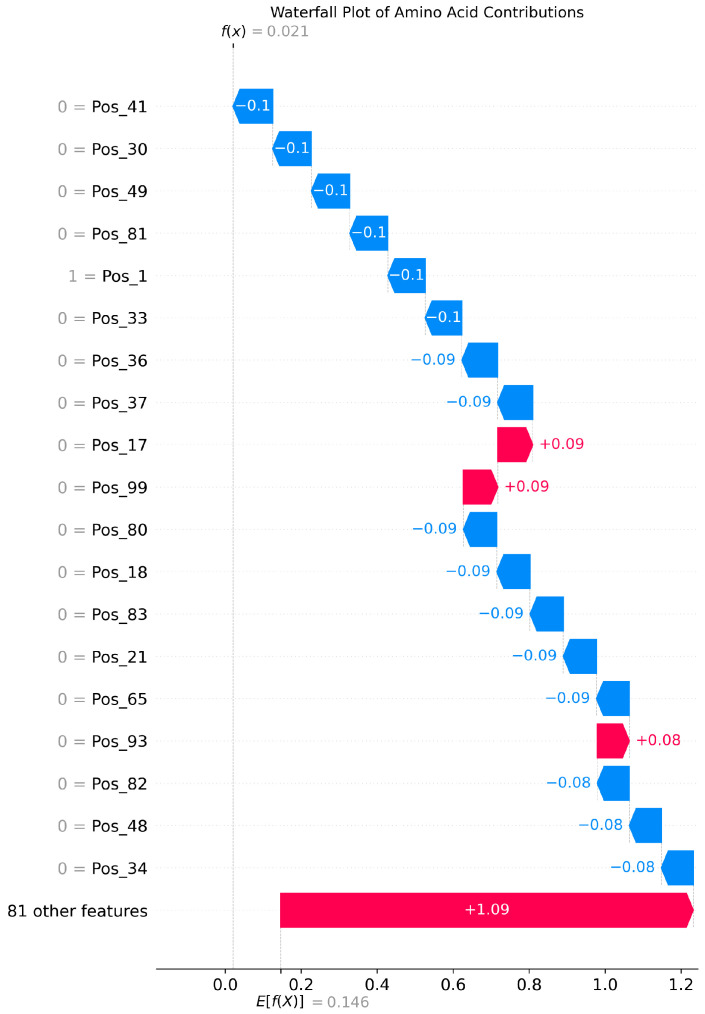
Interpretability analysis of LSTM: feature direction.

**Figure 11:**
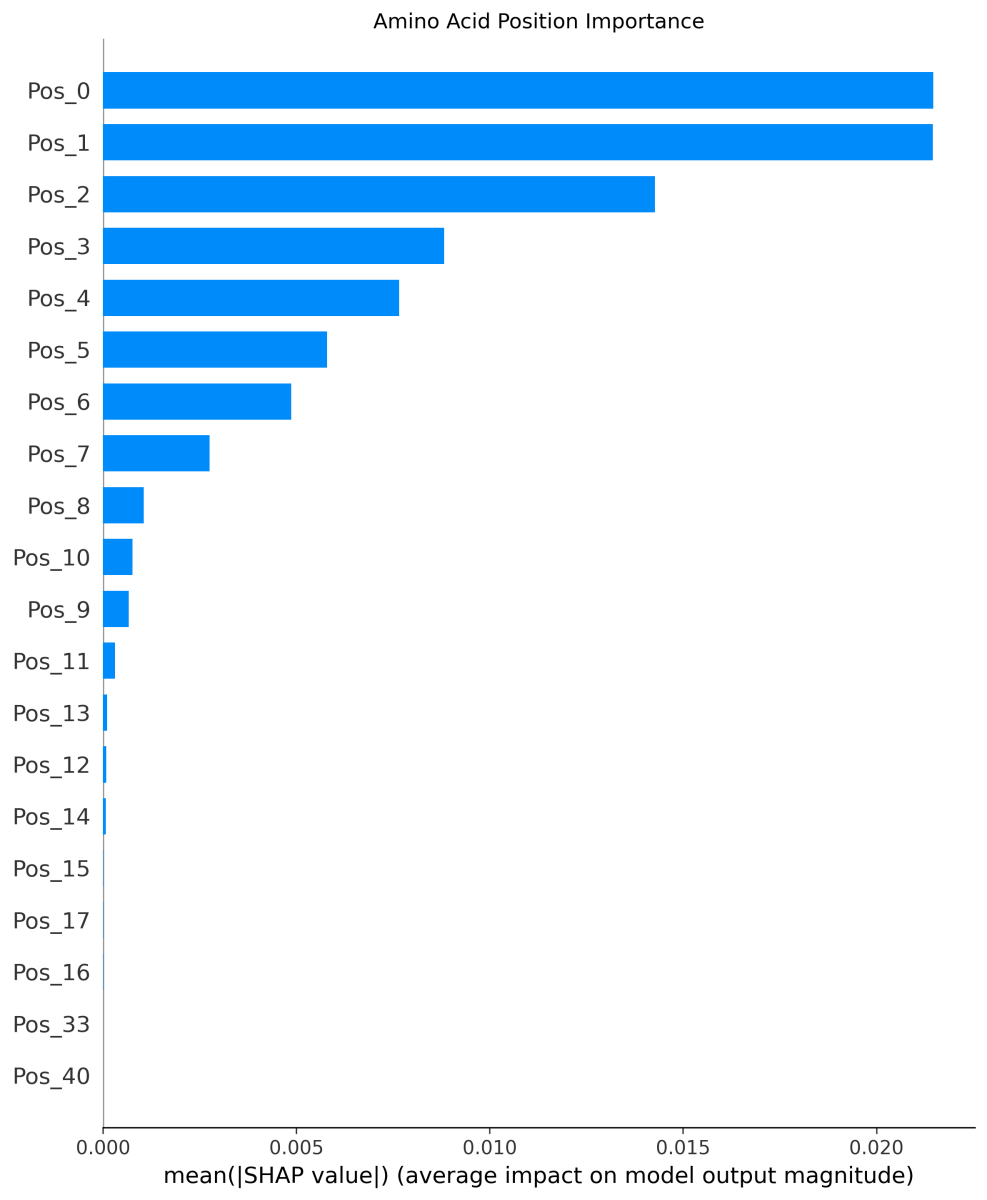
Interpretability analysis of LSTM: global explanation.

Figure 9, Figure 10 and Figure 11 present the interpretability analysis of the LSTM model. For local interpretations, the model had a predicted value of 0.03. In terms of feature direction, the cumulative contribution reached +1.09, indicating that the synergistic positive effects of many features have a significant impact on the prediction results. At the global explanation level, this model was also similar to the previous two models. In summary, the model can not only identify key amino acid positions but can also provide transparent interpretability that supports its positive effectiveness in umami peptide prediction.

### 3.3. Umami Peptide Screening Results

Ten high-probability umami peptide candidates were obtained through integrated model screening: DDSMAATGL, DGEEDASM, ESEGESGK, DEEEVDI, ADEETGA, EEDEAK, DSDVAVAVV, DTAVSTVAQ, EEVDEAR, and EEEEKK (see Table 4). These peptide segments ranked among the top ten in terms of the average prediction probability in the integrated model, and their prediction results were validated by four sub-models (see Table 5 for detailed probabilities of the four sub-models). Further analysis showed that the molecular weight distribution of the candidate peptides ranged from 691.65 to 890.95 Da. DDSMAATGL, DSDVAVAVV, and DTAVSTVAQ showed a positive GRAVY value, indicating they are hydrophobic proteins, while the other seven peptides all showed negative GRAVY values, indicating they are hydrophilic proteins. Charge characterization revealed that the isoelectric points (pI) of these ten peptides ranged from 3.39 to 4.49, with all being negatively charged and less than 7.

According to the toxicity prediction assessment by ToxinPred, none of the ten candidate peptide segments showed potential toxicity risks, thereby meeting the safety requirements for food and drugs. Based on the integrated model evaluation results and physicochemical property analysis, these ten peptides were finally selected for molecular docking validation experiments.

### 3.4. Structural Model of the Umami Receptor T1R1/T1R3

**Figure 12:**
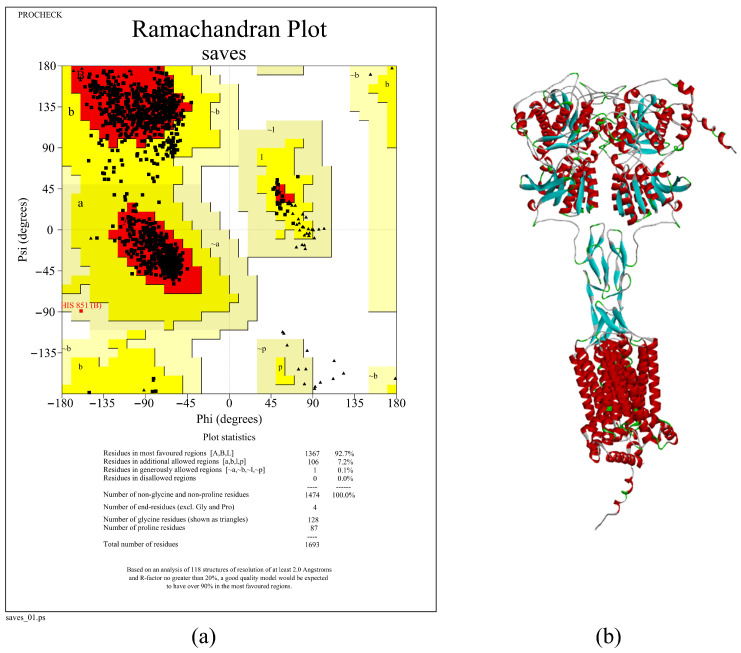
Ramachandran plot and structural model diagram of T1R1/T1R3 receptors: (**a**) Ramachandran plot; (**b**) structural model of receptors.

The T1R1/T1R3 model, which was computationally generated and refined, is shown in Figure 12. Ramachandran plot analysis indicated that the model structure is highly reliable; 100% of residues are located in allowed regions (92.7% in the most favored regions, 7.2% in additionally allowed regions, and 0.1% in generously allowed regions), with no residues in disallowed regions. It can be seen that the receptor model we constructed is structurally complete and can be used for ligand docking and subsequent functional validation studies [49].

### 3.5. Molecular Docking Between Umami Peptides and Umami Receptors

**Figure 13:**
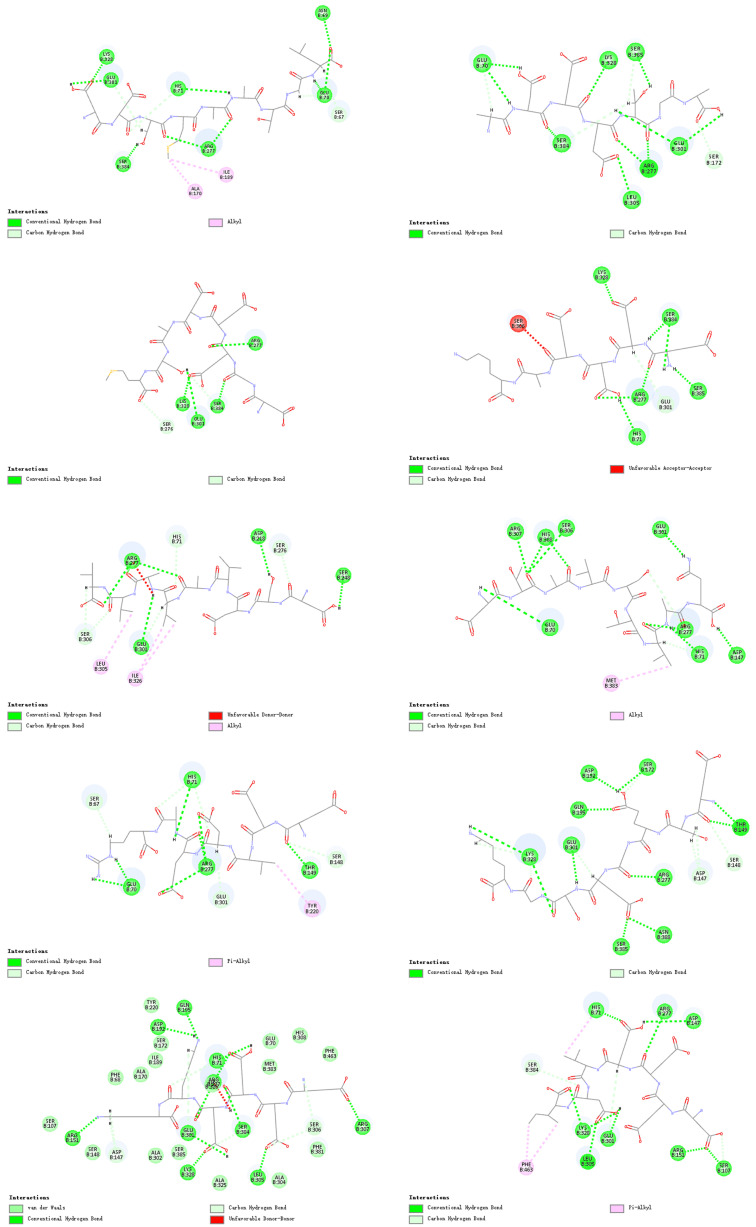
Docking results of T1R1/T1R3 with ten umami peptides.

Figure 13 shows the main active amino acid residues of the umami receptor, including Glu301, Ser384, Lys328, His71, Arg277, Asn69, Glu70, and Ser67. Among them, Glu301 and Arg277 are particularly prominent, as they were present in all tested umami peptides; meanwhile, Lys328 and His71 were found in seven peptides. This distribution pattern directly indicates that Glu, Arg, Lys, and His play a decisive role in umami perception through frequent binding.

Summarizing the above findings, the umami receptor T1R1/T1R3 achieves precise binding with different peptides through multiple molecular interaction modes: hydrogen bonds act as the main driving force, synergistically stabilizing the ligand–receptor complex with C-H bonds, alkyl bonds, and π-alkyl bonds. The high-frequency involvement of key residues such as Glu301, Arg277, Lys328, and His71 reveals the molecular recognition mechanism in umami perception, while the dynamic distribution of binding sites reflects the adaptive regulation of binding modes by ligand structural diversity. These findings provide structural clues for elucidating the molecular basis of umami signal transduction and lay a theoretical foundation for the development of targeted receptor design strategies in food flavor engineering.

Ten umami peptides were docked with the receptor, and the results are shown in Table 6. CDOCKER energy generally refers to the total binding energy between the ligand and the receptor, including interaction energy and ligand internal deformation energy [50]. CDOCKER interaction energy specifically refers to the non-bonded interaction energy between the two, excluding the ligand’s own energy, more directly reflecting binding specificity and stability [51].

Binding energy is the energy released when a ligand binds to a receptor, and the lower values indicate the more stable binding [52]. Stable binding promotes specific interactions between the umami peptide and the umami receptor, thereby triggering the active response and functional expression of the receptor more effectively.

**Figure 14:**
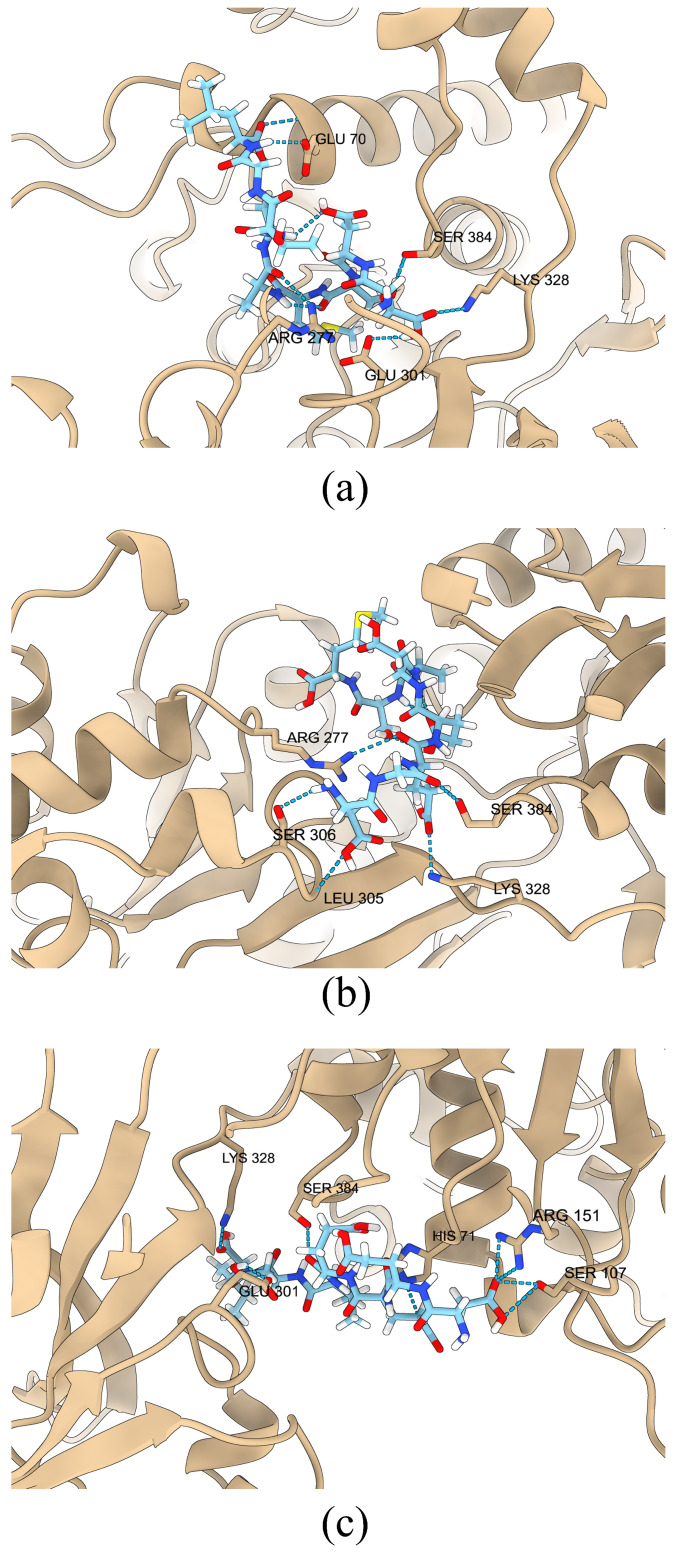
Molecular docking results of three peptides. (**a**) DDSMAATGL, (**b**) DGEEDASM, (**c**) DEEEVDI.

Based on the molecular docking results, we screened out three most promising umami peptides: DDSMAATGL, DGEEDASM, and DEEEVDI. Their molecular docking results are shown in Figure 14. Considering two types of binding energies comprehensively, these three peptides have the lowest CDOCKER energy values, indicating the most stable overall binding conformations. At the same time, their CDOCKER interaction energies are also notably low among all tested peptides, suggesting strong ligand–receptor interaction strength. Therefore, by weighing these two indicators, we ultimately selected these three peptides as the subjects for subsequent research.

### 3.6. Peptide Synthesis and Purification Results

In this study, we successfully synthesized three target umami peptides—DDSMAATGL, DGEEDASM, and DEEEVDI—and obtained high-purity products after purification, with their respective purity values shown in Table 7. These results indicate that the purity of these three peptides has been verified, making them valid for use in subsequent research.

### 3.7. Molecular Dynamics Simulation Results

To analyze the docking results, we also performed 50-nanosecond molecular dynamics simulations on these three peptides to further illustrate the stability of the umami peptides binding to the receptor.

**Figure 15:**
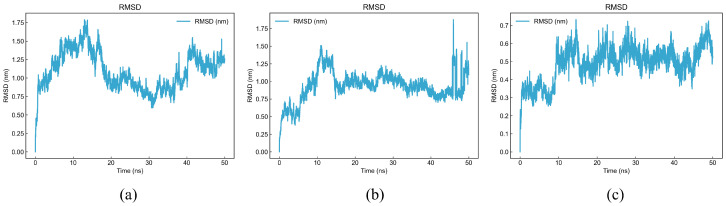
Root mean square deviation curves of three peptides: (**a**) DDSMAATGL, (**b**) DGEEDASM, (**c**) DEEEVDI.

A root mean square deviation plot was obtained to measure the conformational changes of ligand–receptor complexes during post-docking simulations and to assess the stability and reliability of the binding structure; smoother and less fluctuating curves usually represent a more stable system [53]. As shown in Figure 15, the RMSD curve of DDSMAATGL fluctuates less than 0.5 nm after 18 ns, with only a relatively high fluctuation at 15 ns; DGEEDASM fluctuates less than 0.5 nm from 10 ns to 45 ns, with a higher fluctuation only at 47 ns; DEEEVDI remains relatively stable without sudden unstable fluctuations. Therefore, the stability of the three peptides is relatively high.

**Figure 16:**
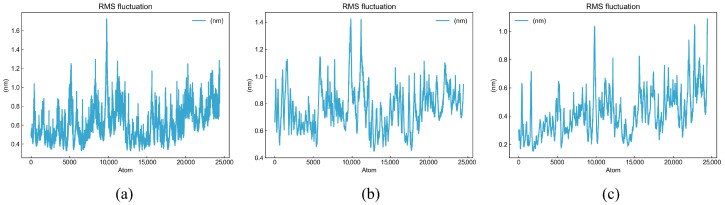
Root mean square fluctuation plot of three peptides: (**a**) DDSMAATGL, (**b**) DGEEDASM, (**c**) DEEEVDI.

The root mean square fluctuation plot reveals the flexibility changes in each residue during the simulation process, with regions of smaller fluctuations usually corresponding to more structurally stable parts [54]. As shown in Figure 16, the fluctuations of DDSMAATGL were basically within 0.8 nm, with higher fluctuations at 5000 and 10,000; DGEEDASM was basically within 1.0 nm, with fluctuations at 10,000; and DEEEVDI was basically within 0.6, with fluctuations at 10,000, 23,000, and 25,000. This indicates that the three peptides are relatively stable in most regions, with small fluctuations, among which DEEEVDI is the most stable.

**Figure 17:**
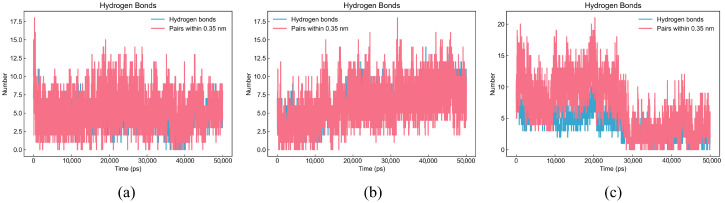
Hydrogen bond plots of three peptides. (**a**) DDSMAATGL, (**b**) DGEEDASM, (**c**) DEEEVDI.

The hydrogen bond plot exhibits the stability of the hydrogen bond network. The blue line represents the actual number of hydrogen bonds, which is the main indicator of stability; meanwhile, the red line represents the number of atomic proximities (distance < 0.35 nm), reflecting potential interactions [55]. If the quantities of the blue and red lines remain stable over time, the system structure is relatively stable. As shown in Figure 17, the red and blue lines of DDSMAATGL and DGEEDASM remain very stable without sudden fluctuations, with the number of hydrogen bonds staying around 10; DEEEVDI remains stable before 30,000 ps, with the number of hydrogen bonds around 7 and, although there are slight fluctuations between 30,000 and 50,000 ps, it also remains stable. Therefore, it can be concluded that the stability of the three peptide structures mainly depends on the hydrogen bonds formed with the receptor.

Based on the above molecular dynamics simulation analysis results, we successfully validated the stability of the predicted binding modes of the three umami peptides with the receptor. These data strongly support that the binding modes of these three umami peptides with the receptor are stable and reliable, providing important molecular-level insights into their mechanism of action.

### 3.8. Sensory Evaluation and Electronic Tongue Results of Synthetic Peptides

In order to investigate the differences in the flavor profiles of different umami peptides, the present study was conducted to jointly analyze the screened peptides using sensory evaluation and the electronic tongue technique.

A lower umami threshold value indicates that less of the substance is required to elicit a detectable sensory response, thereby signifying a stronger taste intensity. As shown in Table 8, all the synthesized peptides possessed both umami and sweet taste. Among them, DDSMAATGL and DEEEVDI exhibited significant fresh–sweet synergistic effects and the best flavor coordination [56]. This was primarily attributed to their low umami thresholds, indicating potent umami intensity, coupled with the absence of any detectable bitter taste. In contrast, DGEEDASM, although exhibiting the strongest sweetness intensity, was accompanied by a slight bitter taste. As shown in Table 9, DGEEDASM had the highest bitterness among the three peptides, and its low saltiness was insufficient to mask this bitterness, thus reducing its overall flavor [57]. The sweetness of all peptides may be due to the fact that the fresh taste receptor shares the T1R3 subunit with the sweet taste receptor, producing a composite taste sensation.

The umami threshold values of the three synthetic peptides from low to high are DDSMAATGL (0.11mg/mL), DGEEDASM (0.37 mg/mL), and DEEEVDI (0.44 mg/mL). Statistical analysis revealed that all three synthetic peptides exhibited highly significant differences in their umami thresholds compared to MSG (0.30 mg/mL) (all *p* < 0.001 ***). Among them, DDSMAATGL (0.11 mg/mL) had a significantly lower umami threshold than MSG, indicating its superior umami intensity. DGEEDASM (0.37 mg/mL) and DEEEVDI (0.44 mg/mL), while having higher thresholds than MSG, also showed a statistically significant difference in their umami intensities compared to MSG. In particular, DDSMAATGL had the lowest umami threshold, representing the highest umami intensity among the tested compounds.

To study the effects of the three peptides on the enhancement of umami taste by MSG, they were dissolved in a 0.35% MSG solution to prepare 1 mg/mL samples, and their regulatory effects on umami perception were systematically evaluated. Experimental data showed that the umami perception thresholds of the three peptides in aqueous solution were 0.11, 0.37, and 0.44 mg/mL, respectively, while in MSG solution, the thresholds significantly decreased to 0.09, 0.27, and 0.31 mg/mL. This indicates that all the synthesized peptides can effectively lower the umami perception threshold, with umami enhancement rates of 18.1%, 27%, and 22.7%. This proves that umami peptides have potential application value in the development of low-sodium seasonings and precise flavor regulation in food.

**Figure 18:**
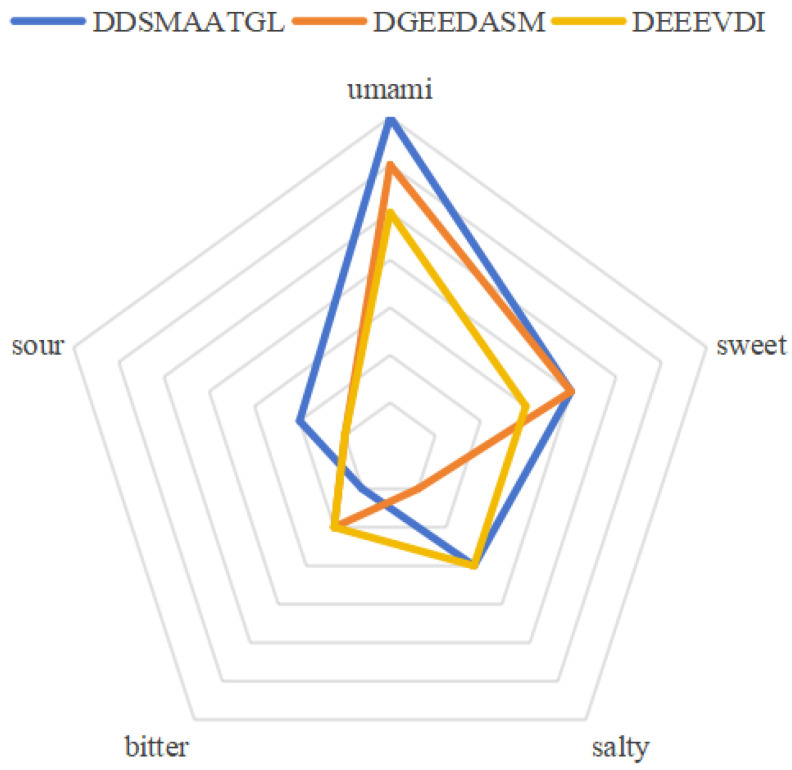
Electronic tongue measurement results of three peptides.

To further verify the taste functional properties of these peptides, this study used electronic tongue technology for analysis and measurement, with the results shown in Table 9 and Figure 18.

The results demonstrate that the main flavors of the three peptides are umami and sweetness, which is consistent with the previous sensory evaluation results presented in Table 8. The ranking for umami was DDSMAATGL > DGEEDASM > DEEEVDI. In terms of sweetness, DDSMAATGL and DGEEDASM scored the same, both being higher than DEEEVDI.

Despite the valuable insights gained from this study, certain limitations should be acknowledged. First, the fermented sausage samples used were the result of a certain production process and of a certain geographical origin. Therefore, our results may not be easily translatable into other types of fermented sausages. Second, this research was limited to three selected umami peptides. Though these peptides produced significant findings, the limited number of studied peptides does not allow us to conclude that our results represent the broader spectrum of umami peptides in fermented sausage or in other food matrices.

## 4. Conclusions

In this study, we utilized metagenomic sequencing data of fermented sausages to explore and construct an integrated model for the prediction of potential umami peptides. The model integrates four sub-models: CNN, LSTM, Attention, and Transformer. Using the constructed model to predict and score the data, we selected the top 10 potential umami peptide sequences with the highest prediction scores. Notably, comprehensive SHAP (SHapley Additive exPlanations) analysis was conducted to elucidate the predictive mechanisms of the integrated model, revealing key amino acid positions and their directional contributions to umami perception.

For further validation, we constructed a Ramachandran plot to assess the structure of the umami receptor T1R1/T1R3, and the results showed that its structure is highly reliable. Then, we performed molecular docking analysis on these top 10 peptides to evaluate their binding ability and stability with the receptor. Based on the molecular docking results, we ultimately selected the three peptides with the lowest binding energy and most stable binding models as candidate umami peptides, namely, DDSMAATGL, DGEEDASM, and DEEEVDI.

The molecular docking results also indicated that amino acid residues Glu301, Arg277, Lys328, and His71 on the receptor play a key role in binding umami peptides through hydrogen bond formation. Further molecular dynamics simulations revealed that the complexes formed by these three peptides with the receptor have good structural stability and efficacy. Subsequently, we conducted sensory evaluations of these three synthetic peptides, all of which exhibited significant umami characteristics.

In summary, in this study, we successfully developed and validated an efficient umami peptide screening strategy based on metagenomic data and an integrated deep learning model. Compared to traditional methods, this approach presents significant advantages in terms of speed, cost-effectiveness, process simplification, and labor savings. This innovative research not only provides powerful computational tools and valuable technical references for the high-throughput discovery of functional peptides (such as umami peptides) but also greatly deepens the understanding of the molecular basis of umami generation through an in-depth analysis of molecular interaction mechanisms. The results of this study lay a solid foundation for the efficient mining of potential functional peptides from complex biological resources in the future, as well as promoting the application of umami peptides in food science and other fields, which has important theoretical significance and broad application prospects.

## Figures and Tables

**Table 1:** Detailed conditions for sausage manufacturing (heating, fermentation, and ripening).

Phase	Time (h)	Temperature (°C)	Relative Humidity (%)
Heating	0–8	20–22	92–95
Fermentation	8–32	19–21	80–88
Fermentation	32–56	19–21	72–84
Fermentation	56–80	18–20	68–76
Fermentation	80–104	17–19	72–78
Fermentation	104–128	16–18	74–82
Fermentation	128–152	15–17	70–78
Ripening	152–960	12–14	74–88

**Table 2:** Model performance metrics.

Model	Precision	Recall	Specificity	Accuracy	F1
CNN	83.7%	80.4%	84.3%	82.4%	0.82
Transformer	81.2%	76.5%	82.4%	79.4%	0.788
LSTM	84.8%	76.5%	86.3%	81.4%	0.804
Attention	84.3%	85.9%	83.7%	81.6%	0.813

**Table 3:** Performance comparison of different models.

Model	Precision	Recall	Specificity	Accuracy	F1
Our Model	83%	79%	84%	81%	0.80
UMpred-FRL [14]	82%	89%	68%	81%	0.85
UmamiYYDS [12]	79%	85%	63%	77%	0.82
UmamiBert [47]	81%	87%	67%	79%	0.84
mlp4umami [48]	82%	84%	70%	79%	0.83

**Table 4:** Prediction of the sequence, physicochemical properties, and toxicity of ten umami peptides selected by the model.

Peptide Sequence	Average Probability	Molecular Weight/Da	PI	GRAVY	Charge	Toxicity
DDSMAATGL	0.9845	879.94	3.56	0.044	-2.1	Non-Toxin
ADEETGA	0.9835	691.65	3.57	-1.143	-3.1	Non-Toxin
DGEEDASM	0.9835	852.82	3.43	-1.438	-4.1	Non-Toxin
EEDEAK	0.9823	719.7	4	-2.683	-3.1	Non-Toxin
DSDVAVAVV	0.9818	873.96	3.56	1.4	-2.1	Non-Toxin
DTAVSTVAQ	0.9818	890.95	3.8	0.311	-1.1	Non-Toxin
EEVDEAR	0.98	846.85	4	-1.786	-3.1	Non-Toxin
ESEGESGK	0.9798	821.80	4.25	-2.1	-2.1	Non-Toxin
EEEEKK	0.9788	790.83	4.49	-3.633	-2.1	Non-Toxin
DEEEVDI	0.9785	847.83	3.39	-1.257	-5.1	Non-Toxin

**Table 5:** Detailed probabilities of the four sub-models and their average values.

Peptide Sequence	CNN	Transformer	LSTM	Attention	Average Probability
DDSMAATGL	0.99	0.998	0.978	0.972	0.9845
ADEETGA	0.985	0.998	0.976	0.975	0.9835
DGEEDASM	0.986	0.996	0.972	0.98	0.9835
EEDEAK	0.982	0.999	0.975	0.973	0.9823
DSDVAVAVV	0.985	0.998	0.977	0.967	0.9818
DTAVSTVAQ	0.987	0.999	0.975	0.966	0.9818
EEVDEAR	0.986	0.999	0.973	0.962	0.98
ESEGESGK	0.985	0.999	0.973	0.962	0.9798
EEEEKK	0.977	0.999	0.971	0.968	0.9788
DEEEVDI	0.973	0.999	0.968	0.974	0.9785

**Table 6:** Docking sites and binding energies of umami peptides and umami receptors.

Peptide Sequence	CDOCKER Energy	CDOCKER Interaction Energy
DDSMAATGL	−117.092	−80.9672
ADEETGA	−105.377	−74.0845
DGEEDASM	−122.913	−83.1365
EEDEAK	−100.164	−63.0616
DSDVAVAVV	−110.689	−71.4591
DTAVSTVAQ	−109.076	−62.2402
EEVDEAR	−117.121	−73.6757
ESEGESGK	−106.384	−87.6919
EEEEKK	−113.611	−86.7792
DEEEVDI	−117.217	−78.1643

**Table 7:** Purity of synthetic peptides.

Peptide Sequence	Purity (%)
DDSMAATGL	99.28
DGEEDASM	99.31
DEEEVDI	99.12

**Table 8:** Taste characteristics and umami thresholds of synthetic peptides.

Peptide Sequence	Taste Description	Threshold Value in Water (mg/mL)	Threshold Value in MSG (mg/mL)
DDSMAATGL	umami, slight sweet, slight salty	0.11	0.09
DGEEDASM	umami, sweet, slight bitter	0.37	0.27
DEEEVDI	umami, slight sweet, slight salty	0.44	0.31

**Table 9:** Electronic tongue measurement results of three peptides.

Peptide Sequence	Umami	Sweet	Salty	Bitter	Sour
DDSMAATGL	7	4	3	1	2
DGEEDASM	6	4	1	2	1
DEEEVDI	5	3	3	2	1

## Data Availability

The original data presented in the study are openly available in https://github.com/fred0504/dataset (accessed on 7 July 2025).
